# Determinants and Improvement of Electrocardiographic Diagnosis of Left Ventricular Hypertrophy in a Black African Population

**DOI:** 10.1371/journal.pone.0096783

**Published:** 2014-05-08

**Authors:** Ahmadou M. Jingi, Jean Jacques N. Noubiap, Philippe Kamdem, Samuel Kingue

**Affiliations:** 1 Department of Internal Medicine and Specialties, Faculty of Medicine and Biomedical Sciences, University of Yaoundé I, Yaoundé, Cameroon; 2 Internal Medicine Unit, Edéa Regional Hospital, Edéa, Cameroon; 3 Centre Médical de la Trinité, Bafoussam, Cameroon; 4 Department of Internal Medicine, Yaoundé General Hospital, Yaoundé, Cameroon; Johns Hopkins University SOM, United States of America

## Abstract

**Background:**

Left ventricular hypertrophy (LVH) is a major cardiovascular risk factor. The electrocardiogram (ECG) has been shown to be a poor tool in detecting LVH due to cardiac and extracardiac factors. We studied the determinants and possibility of improving the test performance of the ECG in a group of Black Africans.

**Methods:**

We studied echocardiograms and electrocardiograms of 182 Cameroonian patients among whom 113 (62.1%) were having an echocardiographic LVH. Echocardiographic LVH was defined as Left Ventricular Mass Indexed to height ^2.7^(LVMI)>48 g/m^2.7^ in men, and >44 g/m ^2.7^ in women or Body Surface Area ≥116 g/m^2^ in men, and ≥96 g/m^2^ in women. Test performances were calculated for 6 classic ECG criteria Sokolow-Lyon, Cornell, Cornell product, Gubner-Ungerleiger, amplitudes of R in aVL, V_5_ and V_6_.

**Results:**

The most sensitive criteria were Cornell (37.2%) and Sokolow-Lyon index (26.5%). The most specific criteria were Gubner (98.6%), RaVL (97.1%), RV_5_/V_6_ (95.7%) and Cornell product (94.2%). The performance of the ECG in diagnosing LVH significantly increased with the severity of LVH for Cornell index (r = 0.420, p<0.0001) and Sokolow index (r = 0.212, p = 0.002). It decreased with body habitus (r = −0.248, p = 0.001) for Sokolow-Lyon index. Cornell index was less affected (age p = 0.766; body habitus: p = 0.209). After sex-specific adjustment for BMI, Cornell BMI sensitivity increased from 37.2% to 69% (r = 0.472, p<0.0001), and Sokolow-Lyon BMI sensitivity increased from 26.5% to 58.4% (r = 0.270, p<0.001).

**Conclusion:**

The test performance of the ECG in diagnosing LVH is low in this Black African population, due to extracardiac factors such as age, sex, body habitus, and cardiac factors such as LVH severity and geometry. However, this performance is improved after adjustment for extracardiac factors.

## Introduction

Increased left ventricular mass (LVM) is an adaptation of the heart to increased hemodynamic burden, and alters cardiac functioning. It is often associated with hypertension and has been shown to be an independent cardiovascular risk factor [1]. Methods of diagnosing left ventricular hypertrophy (LVH) with increasing performance include a comprehensive clinical assessment, chest X-ray, electrocardiography (ECG), Cardiac ultrasound, CT scan, Nuclear Magnetic Resonance imaging (NMR). The ECG is the most cost-effective and is recommended for routine use in assessing LVH [2], [3]. The test performance of the ECG has been shown to be influenced by race, gender, age and body habitus [4], [5]. Adjustment of ECG LVH criteria for body mass index (BMI) and age has been shown to improve the classification accuracy in Caucasians [6]. Such studies are scarce in Sub-Saharan Africa and mostly looked at the test performance of the ECG [7], [8]. Our objective was to study the test performance and determinants of some classic easy to use ECG criteria and the possibility of improvement.

## Methods

### Ethical Considerations

The study was granted ethical approval by the Institutional Review Board of the Health Science Foundation, Cameroon, and was performed in accordance with the guidelines of the Helsinki Declaration. Written informed consent was obtained from all the participants.

### Study Design and Setting

The study was conducted between April and December 2011 in the “Centre Medical de la Trinité”, one of the two cardiologic clinics in the West Region of Cameroon.

### Study Population and Sampling

We included patients aged 18 years or more seen during the study period, consenting to participate, who underwent a Doppler echocardiography and an ECG on the same day. Eligible patients had to fulfil one of these criteria: **i)** normal LV size (Left Ventricular chamber size in end diastole (LVEDd) ≤59 mm and indexed LVEDd to BSA (indexed LVEDd) ≤31 mm/m^2^ in men or ≤32 mm/m^2^ in women) with normal LV or concentric LV remodeling or concentric LV hypertrophy; **ii)** minimally dilated LV (LVEDd ≤59 mm and indexed LVEDd >31 mm/m^2^ in men or >32 mm/m^2^ in women) with normal LV or concentric LV remodeling or concentric LV hypertrophy; **iii)** apparently dilated LV (LVEDd >59 mm and indexed LV ≤31 mm/m^2^ in men or ≤32 mm/m^2^ in women) with normal LV or concentric LV remodeling or concentric LV hypertrophy.

We excluded patients with: **i)** markedly dilated LV, LVEDd >70 mm and indexed LVEDd >31 mm/m^2^ in men or >32 mm/m^2^ in women) as in LV aneurysm and dilated cardiomyopathy; **ii)** chronic obstructive pulmonary disease (COPD); **iii)** septal dyskinesia; **iv)** asymmetric septal hypertrophy (septum/posterior wall ratio >1.3); **v)** technically difficult echocardiography; **vi)** pericardial effusion; **vii)** chest deformity; **viii)** incomplete data (missing weight or height), discordant timing of tests (Echo/ECG).

A sample size of 90 patients was required, assuming the prevalence of 6.25% of LVH among patients with hypertension in Cameroon, a 5% margin of error and a 95% confidence level. The assumption of prevalence of 6.25% among was based on the prevalence of 25% of hypertension in Cameroon [9], [10] and the prevalence of 25% of LVH among patients with hypertension [1].

### Procedure

First, ECG was performed by a trained technician at rest in the supine position with a Mac 500 GE apparatus using standard procedure (speed and voltage regulation of 25 mm/s and 1 mV/10 mm respectively). Secondly, blinded to the ECG, the trans-thoracic echocardiography was performed with the patient in the left lateral decubitus position by an experienced cardiologist using commercially available echocardiography equipment (HP Sonos 2000 Color Doppler ver. A.2, HP Color) and a 4–7 Megahertz transducer.

### Measurements

Subject identity (name, age, sex) were collected by a nurse. The blood pressure (BP) was measured with a mercury sphygmomanometer, the height measured with a stadiometer, and the weight with a clinical scale balance. The body surface area (BSA) was automatically calculated by the echocardiograph to nearest two decimals. The body mass index (BMI) calculated as Weight (kg)/height^2^ (m^2^) to nearest one decimal. Left ventricular measurements were done on long parasternal long axis 2-D guided M-mode using the ASE recommendations [11]. The LVM was calculated using the formula:
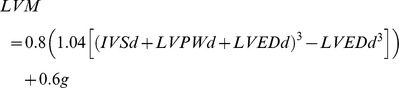



LVM: Left Ventricular Mass

IVSd: Septal thickness in end diastole

LVPWd: Posterior wall thickness in end diastole

LVEDd: Left Ventricular chamber size in end diastole

LVH was defined based on ASE recommendations [11] as Indexed LVM (LVM per m^2^ of BSA or LVM per m^2.7^ of height) >115 g/m^2^ (BSA) or >48 g/m^2.7^ (height in case of obesity) in men and Indexed LVM >95 g/m^2^ or >44 g/m^2.7^ in women. The severity of Left Ventricular Mass (LVM) indexed to BSA was classified according to ASE recommendations as [11]:

- Mild (LVM: 116–132 g/m^2^ in men and 96–109 g/m^2^ in women)
- Moderate (LVM: 133–149 g/m^2^ in men and 110–122 g/m^2^ in women)
- Severe (LVM >149 g/m^2^ in men and 122 g/m^2^ in women).

The relative wall thickness (RWT) was calculated using the formula:
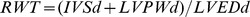



The cut off 0.42 was used to define the LV geometry as:

- Normal: normal LVM and RWT ≤0.42
- Concentric remodeling: normal LVM and RWT >0.42
- Eccentric LVH: LVH and RWT ≤0.42
- Concentric LVH: LVH and RWT >0.42.

The ECG was then interpreted by the same cardiologist (PK). Voltage amplitude was measured to the nearest 0.05 mV and time to the nearest 0.02 s. The following voltage index criteria were evaluated.

- Sokolow-Lyon: SV1+ RV5 or RV6≥3.5 mV
- Cornell voltage index: RaVL+SV3≥2.8 mV in men and 2.0 mV in women
- Cornell product: (RaVL+SV3)×QRSd ≥0.244 mV.s
- Gubner index: RD1+ SD3≥2.5 mV.
- RaVL ≥1.1 mV
- RV5 or RV6≥2.7 mV.

Adjustment for body habitus was done by multiplying the voltage criteria and the BSA or BMI based on previous findings that body habitus has an inverse relationship with ECG voltage criteria.

### Data Analysis

Data was coded, entered and analyzed using the Statistical Package for Social Science (SPSS) version 16.0 for Windows (SPSS, Chicago, Illinois, USA). We described continuous variables using means with standard deviations, and categorical variables using their frequencies and percentages. Pearson’s correlation coefficient was used to associate LVMI (Left Ventricular Mass Index) and the several criteria that were analyzed. Sensitivity and specificity were calculated using 2×2 tables (Classifying ECG LVH as against Echo LVH as the standard). We calculated the specificity as true negatives divided by the sum of true negatives and false positives. Similarly, we calculated the sensitivity as true positives divided by the sum of true positives and false negatives. Regression lines were used to determine cut offs for the BSA or BMI adjusted criteria for two selected voltage criteria (Sokolow-Lyon and Cornell) when the LVM indexed to BSA ≥116 g/m2 for men, and ≥96 g/m2 for women). Mean values of demographic, electrocardiographic and echocardiographic variables were compared using 2-way ANOVA. The chi-square test or its equivalent was used to compare qualitative variables and a *p* value less than 0.05 was considered statistically significant.

## Results

**Figure 1:**
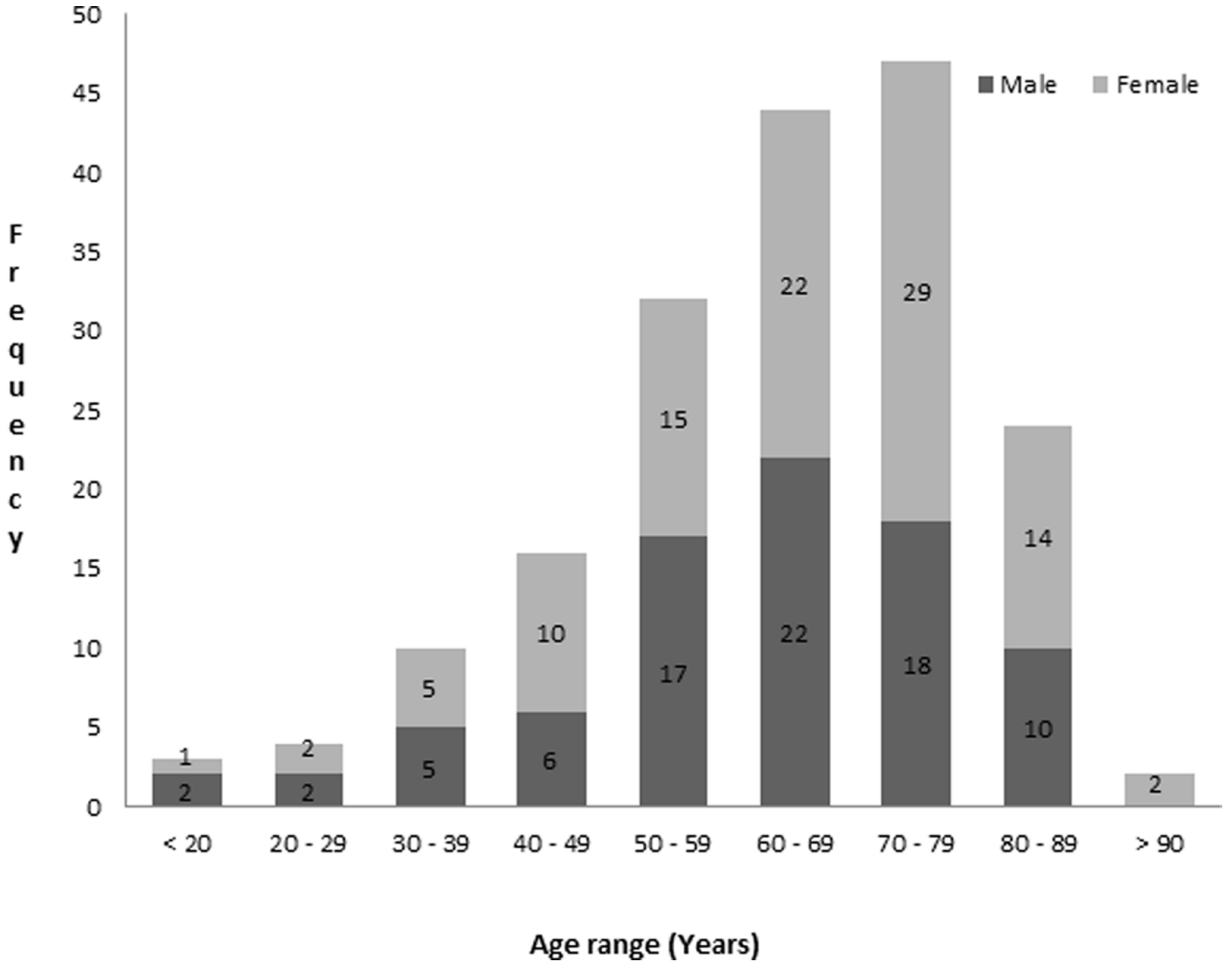
Age distribution of the study population.

**Figure 2:**
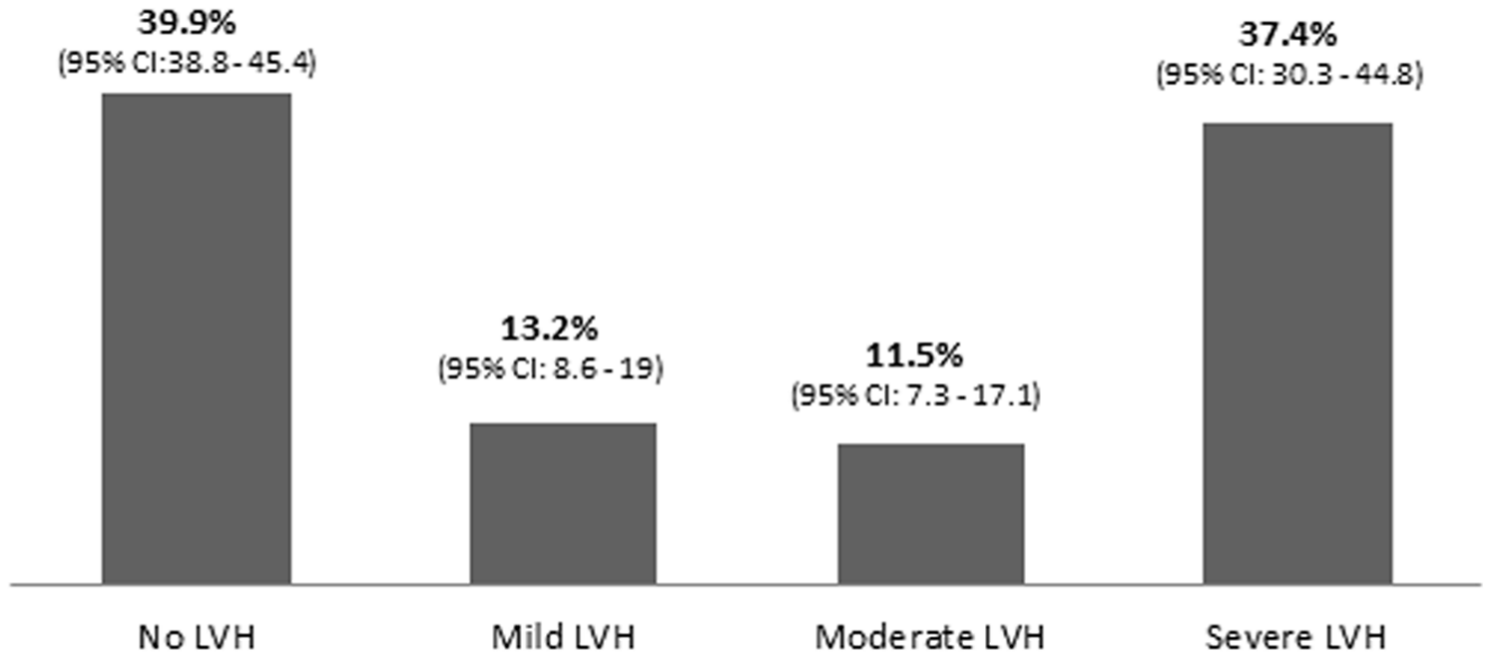
Left ventricular hypertrophy (LVH) severity when the left ventricular mass is indexed to the body surface area.

Clinical, ECG, and echocardiographic data were available and adequate for analysis in 182 patients aged 18 to 99 years with a mean age of 63 years (SD = 15.9). There was no significant age difference between sexes (males 61.7±15.8 years and females 64.1±16 years, p = 0.301). Most of the participants (81.9%) were aged ≥50 years. The most represented age groups were the 70 to 79 years (25.8%) and 60 to 69 years (24.2%) age groups (Figure 1). Fifty four point nine percent of patients (95% CI: 47.4–62) were females and 45.1% (95% CI: 37.7–52.6) were males. The prevalence of echocardiographic LVH was 62.1% (95% CI: 54.6–69.2) for LVM indexed to BSA (LVM ≥116 g/m2 for men, and ≥96 g/m2 for women). The prevalence of LVH when the LVM is indexed to height ^2.7^ (LVM >48 g/m ^2.7^ in men, and >44 g/m ^2.7^ in women) was 72.5% (95% CI: 65.4–78.9%). The concordance between indexing to BSA and height^2.7^ was 87.4% (95% CI: 81.6–91.8). This was comparable between sexes and ages above or below 55 years. The LVH severity when the LVM is indexed to BSA is shown in figure 2.The proportions in the study population and mean values for the different LV geometry types are shown in table 1 and table 2 respectively.

**Table 1:** Clinical, echocardiographic and electrocardiographic criteria (N = 182).

Characteristic	Frequency (%)
Body habitus (BMI)	
Underweight	7 (3.3)
Normal	66 (36.3)
Overweight	55 (30.2)
Obesity	54 (29.7)
Systolic blood pressure >140 mmHg	140 (76.9)
Diastolic blood pressure >90 mmHg	133 (73.1)
LV Chamber size	
Dilated	35 (19.2)
Normal	147 (80.8)
Pulmonary pressure >30****mmHg	61 (35.5)
LVH*	113 (62.1)
LVH**	132 (72.5)
LV Geometry	
Normal	13 (7.1)
Concentric remodeling	43 (23.6)
Concentric hypertrophy	96 (52.7)
Eccentric hypertrophy	30 (16.5)
Ejection fraction >50%	116 (63.7)
Heart rhythm	
Sinus	172 (94.5)
Atrial fibrillation	10 (5.5)
Sokolow LVH	44 (22.5)
Cornell LVH	51 (28)
Cornell product LVH	27 (14.8)
Gubner LVH	11 (6)
RaVL LVH	16 (8.8)
RV_5_/RV_6_ LVH	6 (3.3)

*(LVM Indexed to BSA ≥116 g/m^2^ in men and ≥96 g/m^2^ in women).

**(LVM Indexed to Height ≥49 g/m^2.7^ in men and ≥45 g/m ^2.7^ in women).

**Table 2:** Clinical, electrocardiographic and echocardiographic characteristics comparing mean values relative to the left ventricular geometry type.

Characteristics	Normal N = 13	Concentricremodeling N = 43	Concentric hypertrophy N = 96	Eccentric hypertrophy N = 29	*p* value	Total	Range
**Clinical**							
Age (SD) years	49.9 (19.4)	60.1 (18.1)	66(13.7)	62.9(14.9)	0.003	62.98(15.9)	18–99
Systolic BP (SD)mmHg	147.9(24.6)	155.6(30.4)	169.5(33.1)	146.6(30.7)	<0.0001	160.45(33)	90–260
Diastolic BP(SD)mmHg	92.6(17.2)	94.3(15.9)	99(20.8)	89.2(19.5)	0.09	95.8(19.5)	50–177
BMI (SD) g/m^2^	27.1(6.3)	26.8(5.7)	27.9(5.8)	25.7(5.6)	0.315	27.2(5.8)	16.3–43
BSA (SD) m^2^	1.94(0.38)	1.89(0.26)	1.92(0.32)	1.84(0.32)	0.68	1.9(0.3)	1.27–2.89
**Electrocardiogram**							
Heart rate (SD): beats/min	75.5(11.4)	80.7(21)	76(19.1)	87.3(24)	0.057	78.9(20.3)	45–150
P wave duration (SD) s	0.1(0.01)	0.1(0.02)	0.1(0.02)	0.11(0.02)	0.283	0.10(0.02)	0.08–0.14
P-R (SD) s	0.17(0.03)	0.17(0.02)	0.17(0.03)	0.18(0.03)	0.428	0.17(0.03)	0.12–0.24
QRS							
Duration (s)	0.09 (0.02)	0.08(0.01)	0.08(0.01)	0.10(0.03)	<0.0001	0.08(0.02)	0.06–0.16
Axis	20.3(37.8)	31.5(42.6)	19.3(32)	10.4(36.1)	0.114	20.9	−46–120
QTc (s)	0.39(0.02)	0.39(0.03)	0.39(0.03)	0.38(0.04)	0.004	0.39 (0.03)	0.32–0.48
RaVL (mV)	0.54(0.26)	0.43(0.32)	0.65(0.42)	0.73(0.43)	0.004		
Sokolow (mV)	2.73(0.7)	2.55(0.79)	2.96(0.87)	3.02(0.97)	0.038	28.7(8.7)	6–55)
Cornell (mV)	1.36(0.58)	1.49(0.54)	1.95(0.72)	2.65(0.88)	<0.0001	19.2(8)	4–42
Cornell product (mV.s)	0.113(0.042)	0.115(0.045)	0.161(0.07)	0.261(0.129)	<0.0001	1.64(0.91)	0.32–6.72
Gubner - Ungerleiger(mV)	1.07(0.41)	0.96(0.56)	1.36(0.77)	1.51(0.79)	0.713	12.7(7.3)	0–42
**Echocardiogram**							
LA (cm)	3.6(0.8)	3.3(0.6)	4.1(0.9)	4.6(0.8)	<0.0001	3.97(0.88)	1.92–6.56
LVPWd (cm)	0.95(0.12)	1.09(0.13)	1.32(0.21)	1.15(0.15)	<0.0001	1.21(0.21)	0.79–2.2
IVSd (cm)	0.92(0.12)	1.17(0.12)	1.41(0.19)	1.15(0.15)	<0.0001	1.28(0.23)	0.74–2.11
DTDVg Index (cm/m^2^)	2.59(0.3)	2.15(0.4)	2.49(0.5)	3.48(0.5)	<0.0001	2.58(0.63)	1.39–4.62)
EF (%)	63.6(12.3)	63.4(12.1)	57(13.3)	37.5(14.4)	<0.0001	55.76(15.6)	14–88
LVM Index (g/m^2^)	80.72(16)	80.95(16.9)	136.26(38.2)	178.68(43.97)	<0.0001	126.22(48)	52.4–280.6
RVSP mmHg	34(10.7)	38.2(15.9)	43.9(17.6)	44.2(20.6)	0.485	42.3(17.5)	14–90

### Determinants of Test Performance

Table 3 shows the ECG voltage test performances with the severity of LVH. The sensitivity increased with the degree of LVH without a major decrease in the specificity. This was especially seen with the Cornell voltage criterion. Table 4 shows some other possible factors that could determine the test performance of the Sokolow-Lyon and Cornell voltage indices. The Sokolow-Lyon criterion is more sensitive in males though to a lesser extent than the Cornell criterion, which is more sensitive in females. Overall, Cornell voltage criterion performed better in both sexes. The test performance of the Sokolow-Lyon criterion significantly decreased with age (r = −0.159, p = 0.032), while the Cornell criterion showed a non-significant increase with age (r = 0.082, p = 0.766). The body habitus generally showed a significant negative effect on the Sokolow-Lyon criterion (r = −0.248, p = 0.001) while the Cornell criterion showed a non-significant positive effect (r = 0.094, p = 0.209). A positive effect was shown with the LV geometric pattern concordant with the pathologic evolution in both the Sokolow –Lyon and Cornell criteria.

**Table 3:** Sensitivity and specificity at different thresholds for Left Ventricular Hypertrophy Mass Indexed to Body Surface Area (ASE).

Index	Mild LVH* (N = 24)		Moderate LVH** (N = 21)		Severe LVH*** (N = 68)		r	*p* value
	Sensitivity	Specificity	Sensitivity	Specificity	Sensitivity	Specificity		
Sokolow-Lyon	26.5%	84.1%	27.8%	82.6%	28.4%	80.9%	0.212	0.004
Cornell	37.2%	87%	42.2%	85.9%	49.3%	84.3%	0.420	<0.0001
Cornell product	20.4%	94.2%	23.3%	93.5%	31.3%	94.8%	0.419	<0.0001
Gubner-Ungerleiger	8.8%	98.6%	6.7%	93.5%	6%	93.9%	0.165	0.027
RaVL	11.5%	95.7%	4.9%	91.3%	10.4%	92.2%	0.179	0.016
RV_5_/V_6_	3.5%	97.1%	1.6%	97.8%	4.5%	97.4%	0.082	0.269
All combined	55.8%	73.9%	60%	70.7%	67.2%	68.7%	NA	NA

Mild LVH*: For at least LVM ≥116 g/m^2^ for men and ≥96 g/m^2^ for women.

Moderate LVH**: For at least LVM ≥133 g/m^2^ for men and ≥110 g/m^2^ for women.

Severe LVH***: For at least LVM ≥149 g/m^2^ for men and ≥122 g/m^2^ for women.

**Table 4:** Factors influencing the specificity and sensitivity for two selected index criteria: Sokolow-Lyon and Cornell indices.

Index	Sokolow-Lyon				Cornell			
	Sensitivity	Specificity	r	*p* value	Sensitivity	Specificity	r	*p* value
**Sex**								
Male	27.6%	83.3%	NA	NA	23.1%	90%	NA	NA
Female	24.3%	88.5%	–		49.2%	84.6%	–	–
**Age**								
≤55 years	35%	82.6%	–0.160	0.032	47.2%	87.1%	0.082	0.766
>55 years	26%	88.9%	–	–	32.5%	86.8%	–	–
**Body habitus**								
Underweight	40%	–	–0.248	0.001	20%	–	0.094	0.209
Normal	31.8%	72.7%	–	–	27.3%	86.4%	–	–
Overweight	29%	91.7%	–	–	45.2%	83.3%	–	–
Obesity1	15.6%	90.9%	–	–	28.1%	90.9%	–	–
**LV geometry**								
Normal	–	84.6%	0.172	0.020	–	100%	0.472	<0.0001
Remodeling	–	87.4%	–	–	–	87.8%	–	–
Concentric LVH	25.9%	73%	–	–	30.9%	73.4%	–	–
Eccentric LVH	30%	–	–	–	56.7%	–	–	–

NA: not applicable.

### Test Performances after Adjusting for Sex and Body Habitus

Table 5 shows the test performances of the un-adjusted and adjusted Sokolow-Lyon and Cornell indices with thresholds determined from regression equations at LV mass index ≥116 g/m^2^ in males and ≥96 g/m^2^ in females. The sensitivity doubled with both criteria with a decrease in the specificity by half and by a third with both the Sokolow-Lyon and Cornell criteria respectively. The test accuracy was better in the adjusted criteria.

**Table 5:** Test performance of unadjusted and adjusted indices when the Left Ventricular Mass is indexed to the Body Surface Area.

Index	Threshold(Male/Female)	Sensitivity	Specificity	PPV	NPV	Accuracy	Likelihood ratio	r	*p* value
**Sokolow-Lyon**									
Unadjusted (mV)	3.5	26.5%	84.1%	73.2%	41.1%	48.4%	1.66	0.162	0.029
Adjusted to BSA (mV.m^2^)	5.17/5.1	55.8%	46.4%	63%	39%	52.2%	1.04	0.283	<0.001
Adjusted to BMI (mV.Kg/m^2^)	72.82/71.49	58.4%	40.6%	61.7%	37.8%	51.7%	0.98	0.270	<0.001
**Cornell**									
Unadjusted (mV)	2.8/2.0	37.2%	87%	82.4%	45.8%	56%	2.86	0.424	<0.0001
Adjusted to BSA (mV.m^2^)	3.11/3.02	68.1%	52.2%	70%	50%	62.1%	1.43	0.501	<0.0001
Adjusted to BMI (mV.Kg/m^2^)	44.23/42.74	69%	49.3%	69%	50%	61.5%	1.36	0.472	<0.0001
**Combined indices**									
Unadjusted	NA	50.4%	76.8%	78.1%	48.6%	60.4%	2.08	NA	NA
Adjusted to BSA	NA	77%	38.1%	62.1%	38.1%	56.6%	1.24	NA	NA
Adjusted to BMI	NA	79.6%	23.1%	62.9%	39.5%	57.7%	1.04	NA	NA

PPV: positive predictive value; NPV: negative predictive value; NA: not applicable.

## Discussion

These findings demonstrate that the test performance of the ECG in diagnosing LVH is low in this Black African population. This is affected by a number of extracardiac factors such as age, sex, body habitus, and cardiac factors such as LVH severity and geometry. Sex specific adjustment to body habitus improves the test sensitivity. Dzudie et al. reported a similar finding of performance in a similar population [7]. Dada et al. reported a high test performance in a neighboring community [8]. The poor performance of the ECG in detecting LVH has been shown in several studies [4], [12], [13], [14].

### ECG and Sex

Sex is a major determinant of body structure and function. The Sokolow-Lyon criterion performed better in males. This could be attributed to the fixed voltage criterion that does not take account of structural body difference. Levy et al. reported an overall better performance in males where most indices used are non-sex specific [4]. Cornell criterion had an overall better performance especially in females. Rodrigues et al. reported a similar finding [14].

### ECG and Age

Sokolow-Lyon and Cornell indices test performance decreased with advancing age. This was particularly significant with the Sokolow-Lyon criterion. The Cornell criterion showed a very weak but non-significant positive trend with age. Left axis shift and precordial voltages have been shown to decrease with age in men and left axis shift in women [15]. This could explain the trend observed with both criteria. Sokolow-Lyon index depends only on precordial leads while Cornell index depends on a precordial and limb leads. Left axis shift might lead to a higher R voltage in the augmented unipolar left limb lead used in Cornell index, thus a positive but non-significant trend. Aging is associated with physical changes such as a reduction in height. The enlarged heart then deviates to the left with changes in the cardiac vector as explained by Levy et al. [15]. Voltage changes associated with age cancel out with the Cornell index, thus less affected by age. The overall lower sensitivity with age could be attributed to lower tissue conductance, a point for further investigations.

### ECG and Body Habitus

Sokolow-Lyon index showed a significant negative trend with increasing body size while Cornell index showed a non-significant positive trend. Levy et al. reported an overall negative trend [4]. Compared with normal weight patients, overweight and obese patients had a higher sensitivity and specificity with the Cornell index. This shows that the Cornell index is more accurate in diagnosing LVH in overweight and obese patients than Sokolow-Lyon index. Okin et al. reported a similar finding [5], [16]. This could be explained by the high prevalence of LVH with obesity and attenuating effects of obesity on precordial voltages [17]. The less affected augmented limb lead in Cornell index will pull the voltage towards higher values for LVH. Sokolow-Lyon criterion depends on the precordial leads that undergo significant attenuation in overweight and obese patients, thus a decrease in its sensitivity.

### ECG and LV Geometry

The sensitivity progressively increased with the severity of LVH and with the pathologic evolution of the LV with both indices. This was higher with the Cornell index. Levy et al. and Schillaci et al. reported a similar finding of increased sensitivity with LVH [4], [13].

### Unadjusted and Adjusted Criteria

Sex specific adjustment to body habitus markedly increased the sensitivity by 2 fold with both Sokolow-Lyon and Cornell indices, with a fall in the specificity by a third and by half with Cornell and Sokolow-Lyon criteria. The highest increase was noted with Cornell index or combined indices adjusted to body mass index. With a fall in specificity, the predictive values were comparable (Table 5). Okin et al. and Norman et al. reported similar effects [5], [6]. We used thresholds estimated from regression equations, which are considered to be the mean in the measured parameters. Sensitivity and specificity move in opposite directions [18]. Specificity increases with the set threshold, with a concomitant decrease in the sensitivity. Concomitant high sensitivity and specificity are seen in measurements with less spread about the mean (very strong correlation). This is however difficult to achieve with biological measurements especially when reproducibility is not perfect. Biological parameters have a high variability and are less reproducible. This gives sensitivities and specificities wide apart. The cut-off point between normal and abnormal of a test may therefore be varied to increase sensitivity or specificity (with concomitant decrease in the other) according to what we are using the test for [19]. LVH has been shown to be an independent cardiovascular risk factor and as well treatable [1], [16]. It is best not to miss the diagnosis thus, the need for a test with a high sensitivity as the sex specific adjusted criteria [19]. Rodrigues et al. proposed a lower sex specific threshold for the Cornell index with the aim of improving the sensitivity [14]. This will be particularly useful in high risk patients. Further analysis using the ROC curve will give a clearer view of the situation with optimal cut-offs.

One of the limitations of our study is that only a single observer made the diagnosis of LVH both in the echocardiography and ECG. Echocardiography measurements are observer-dependent and no agreement test was carried out. The study was a specialist clinic based with selection bias of patients with heart disease. There is the risk of generalizing our findings. The echocardiography was used for comparison instead of more accurate tools like the CT scan and MRI. These are however very expensive, highly technical and not widely available. The echocardiography is widely available and user friendly, and has been shown to have a strong correlation with autopsy findings [20]. Not all possible determinants of electrocardiographic diagnosis of LVH were studied such as the cardiovascular risk factors and ethnicity. Moreover, our study is limited by the small number of subjects aged less than 40 years, as there is a significant difference in voltage between this younger age group and the more elderly. Hence the relationship between age and ECG test performance should be considered with caution. Notwithstanding, our work has some implications. We have contributed in paving the way for the ECG to be more accurate, since it is widely available and more user friendly than the echocardiography, and more accurate than the clinical method and X-ray in diagnosing LVH. Similar studies need to be carried out in the general population. A combined adjustment for age, sex, and body habitus with optimal cut-offs derived from the ROC curve is necessary for a clearer view of the possibility of improving the ECG. Fewer ECG criteria were studied further. Our choices were motivated by their better performance and are easier to use in routine clinical practice.

## Conclusion

These findings demonstrate that the test performance of the ECG in diagnosing LVH is low in this black African population. This is affected by a number of extracardiac factors such as age, sex, body habitus, and cardiac factors such as LVH severity and geometry. Cornell voltage criteria had a higher performance and less affected than the Sokolow-Lyon voltage criteria. Sex specific adjustment to body habitus improves the test sensitivity by two fold and a fall in the specificity.
